# Development of Novel Microsatellite Markers for the Secret Cave Cricket, *Ceuthophilus secretus*

**DOI:** 10.1673/031.013.6401

**Published:** 2013-06-25

**Authors:** Nichole Hutchison, Paul. L. Leberg, Richard F. Lance

**Affiliations:** 1 Department of Biology, University of Louisiana at Lafayette, Lafayette, LA 70504, USA; 2 Environmental Processes Branch, Environmental Laboratory, US Army Engineer Research & Development Center, Vicksburg, MS 39180, USA

**Keywords:** codominant markers, population genetics, simple sequence repeat DNA, trogloxenes

## Abstract

The secret cave cricket, *Ceuthophilus secretes* Scudder (Orthroptera: Rhaphidophoridae), is an obligate trogloxene endemic to central Texas, USA, and is a primary source of energy and nutrients for sensitive cave ecosystems. In this study, nine polymorphic microsatellite markers were developed from genomic DNA of *C. secretes.* Genotypes of 120 individuals sampled from four localities on the Fort Hood Military Reservation, Killeen, Texas, were analyzed to characterize the polymorphism at each locus. The number of alleles ranged from 19 to 46. All paired loci were in linkage equilibrium. Four loci had significant deviations from Hardy-Weinberg equilibrium. Observed heterozygosity varied from 0.793 to 0.959. These loci provide a means of characterizing population genetic structure in this species.

## Introduction

The Edwards Plateau of central Texas is one of the largest continuous karst regions in the USA, subsidence sinkholes and caves being the major karst features of this area (Venni 1994). The many small caves located throughout this region contain unique ecosystems, with some harboring a variety of rare endemics, including several endangered invertebrates (U.S. Fish and Wildlife Service 1988, 1993, 2000). Many of these species are troglobites that do not leave the cave environment. The secret cave cricket, *Ceuthophilus secretus* Scudder (Orthroptera: Rhaphidophoridae), which is found in many cave ecosystems in central Texas, is considered to be a trogloxene (Taylor et al. 2005; Lavoie et al. 2007) because it forages on the surface at night, but typically returns to the cave for other parts of its daily activities. Energy and nutrients, in the form of *C. secretus* biomass, feces, and eggs, are thereby transferred from the surface into the cave ecosystem, where they are utilized by various predators and scavengers and become part of cave nutrient and bioenergy cycles (Taylor et al. 2007). Given the species' importance to the cave ecosystems, it is considered a keystone species on which many of troglobites depend (Taylor et al. 2005; Lavoie et al. 2007).

Genetic markers are largely lacking for the family Rhaphidophoridae, excepting five microsatellite DNA loci described for use in the genus *Dolichopoda* (Bernardini and Ketmaier 2002). Nine new microsatellite DNA loci that can be used in characterizing population genetic structure in *C. secretus* were developed in this study. These markers should assist researchers in characterizing gene flow, and general dispersal and colonization patterns, within and among *C. secretus* populations.

Landscape and connectivity (dispersal, migration, gene flow) are likely important features of *C. secretus* natural history, as evidenced by the species' highly variable residency and abundance in different caves over time (C. Pekins, Environmental Division, US Army Garrison-Fort Hood personal communication). Therefore, the ability to use genetic data to elucidate connectivity among caves (or populations of multiple caves) and to identify landscape features that influence connectivity should prove useful for the management and conservation of this critical player in cave ecology.

## Materials and Methods

Whole genomic DNA was extracted from the legs of each *C. secretus* specimen using a DNeasy Blood and Tissue Kit following the manufacturer guidelines (Qiagen, www.qiagen.com). By utilizing the DNA from eight individuals, Genetic Identification Services (GIS; www.genetic-id-services.com) was able to develop four enriched microsatellite genomic libraries. These libraries were enriched for CA, AAC, ATG, and TAGA motifs. GIS designed polymerase chain reaction (PCR) primers for 77 microsatellite-containing clones using Designer PCR version 1.03 (Research Genetics, Inc., www.lifetechnologies.com). From this pool of potential loci, reaction conditions were optimized for nine loci showing evidence of polymorphism.

Microsatellite amplifications were performed in 10 µl reaction volumes with 1.0 µl of DNA template in a solution of 0.18 mM of each primer, 1 X NH_4_ PCR Reaction Buffer (Bio-line, www.bioline.com), 0.80 mM dNTPs, 0.025 units of Biolase DNA polymerase (Bioline), and 0.25 mM MgCl_2_. All PCRs were performed in a MyCyler Thermal Cycler (Bio-Rad, www.bio-rad.com) under the following conditions: initial incubation at 94° C for 3 minutes, followed by 30 cycles of 94° C for 40 seconds, 54° C to 59° C (according to each primer annealing temperature; see Table 1) for 40 seconds, and 72° C for 30 seconds, followed by a final 10 minute incubation at 72° C.

In order to characterize microsatellite loci, 30 individuals from each of four populations (N = 120) located in the Fort Hood Military Reservation near Killeen, TX, USA, were genotyped. The four populations, from Geocache Cave, Lost Chasm Cave, New Cave, and Sanford Pit Cave, were located on four different limestone ridges and separated by an average distance of 18.8 km (SD = 11.3 km). Individuals were genotyped using an ABI 3130 Genetic Analyzer with labeled forward primers (6-FAM, NED, HEX) and ROX 400 size standard (Applied Biosystems, www.appliedbiosystems.com). Each locus was run singly on the genetic analyzer. All loci were tested for linkage disequilibrium and deviations from Hardy-Weinberg expectations using GENEPOP 4.1.0 (Rousset 2008). A sequential Bonferroni correction (Rice 1989) was applied to correct type I error rates for multiple significance tests. Observed and expected heterozygosities were calculated by using GENALEX 6.0 (Peakall and Smouse 2006). GENEPOP was used to test for significant differentiation in allele frequencies among populations and to calculate interpopulation FSTs.

## Results

Genotyping results are summarized by locus in Table 1. All nine loci were polymorphic, with numbers of alleles ranging from 19 to 46. Observed heterozygosity ranged from 0.793 to 0.959, while expected heterozygosity values ranged from 0.854 to 0.969. Differences between observed and expected heterozygosity were due, at least in part, to having estimated heterozygosity using data pooled from four genetically differentiated populations (the Wahlund effect). Four of nine loci showed significant deviations from Hardy-Weinberg equilibrium (HWE) in at least one population. MICROCHECKER 2.2.3 (Van Oosterhout et al. 2006) analysis suggested that the presence of null alleles might explain heterozygote deficiencies for Cese2 (H_E_ = 0.957, H_O_ = 0.704, null allele frequency (Null_F_) = 0.132) and Cese9 (H_E_ = 0.910, H_O_ = 0.704 Null_F_ = 0.108) in POP1, for loci Cesel (H_E_ = 0.952, H_O_ = 0.844, Null_F_ = 0.057) and Cese3 (H_E_ = 0.931, H_o_ = 0.719, Null_F_ = 0.115) in POP2, and for locus Cese2 (H_E_ = 0.945, H_O_ = 0.793, Null_F_ = 0.081) in POP4. There were no cases of linkage disequilibrium among loci. All allele frequencies were significantly different between pairs of populations (*p* ≤ 0.009), and interpopulation FSTs ranged from 0.001 to 0.014.

## Discussion

In this study, a suite of microsatellite DNA markers were developed for the secret cave cricket, *C. secretus.* These markers should provide insight into genetic diversity, gene flow, and landscape genetics in a species for which little such information is available. In a species such as *C. secretus,* which resides in a habitat that is difficult to access, with highly unpredictable presence and abundance among caves and over time, and that is largely nocturnal and secretive (hiding under rocks and debris) when on the surface, molecular techniques likely provide the best means for determining colonization and dispersal patterns.

Four of nine microsatellite markers showed genotypic frequencies that deviated from those frequencies expected under HWE. Population substructure, inbreeding, or technical artifacts (e.g., null alleles) could be the cause for these deviations. Additional populations should be examined to determine if these loci consistently show evidence of null alleles and deviations from HWE. No evidence of deviations from HWE expectations for the other five loci was found. Although care should be used when including loci with null alleles in some analyses, the resulting bias is often small (Carlsson 2008), and methods exist allowing their inclusion in several estimators of genetic parameters (van Oosterhout et al. 2004; Chapuis and Estoup 2007).

**Table 1.**
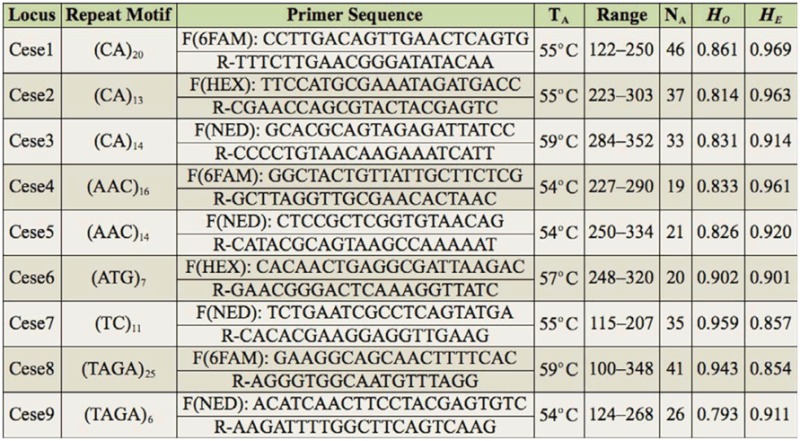
Locus name followed by repeat motif present in sequenced fragment, primer sequences with designated fluorescent dye, primer annealing temperature (T_A_), and size range of alleles in base pairs (range). Also, number of alleles (N_a_), observed heterozygosity (H_o_), and expected heterozygosity (H_E_) based on combined data from four populations of *Ceuthophilus secretus* are reported. Differences in Ho and HE can be at least partly explained by the Wahlund Effect.

## Abbreviations

**HWE**,: Hardy-Weinberg Equilibrium
**PCR**,: polymerase chain reaction

## References

[bibr01] Barr TC (1968). Cave ecology and the evolution of troglobites.. *Evolutionary Biology*.

[bibr02] Bernardini C, Ketmaier V (2002). Isolation and characterization of five microsatellite loci in *Dolichopoda* cave crickets (Orthoptera: Rhaphidophoridae).. *Molecular Ecology Notes*.

[bibr03] Caccone A (1985). Gene flow in cave arthropods: A qualitative and quantitative approach.. *Evolution*.

[bibr04] Carlsson J (2008). Effects of microsatellite null alleles on assignment testing.. *Journal of Heredity*.

[bibr05] Chapuis M-P, Estoup A (2007). Microsatellite null alleles and estimation of population differentiation.. *Molecular Biology and Evolution*.

[bibr06] Lavoie KH, Helf KL, Poulson TL (2007). The biology and ecology of North American cave crickets.. *Journal of Cave and Karst Studies*.

[bibr07] Peakall R, Smouse PE (2006). Genalex 6: genetic analysis in Excel. Population genetic software for teaching and research.. *Molecular Ecology Notes*.

[bibr08] Rice WR (1989). Analyzing tables of statistical tests.. *Evolution*.

[bibr09] Rousset F (2008). Genepop'007: a complete reimplementation of the Genepop software for Windows and Linux.. *Molecular Ecology Resources*.

[bibr10] Taylor SJ, Krejca JK, Denight ML (2005). Foraging range and habitat use of *Ceuthophilus secretus* (Orthoptera: Rhaphidophoridae), a key trogloxene in central Texas cave communities.. *American Midland Naturalist*.

[bibr11] Taylor SJ, Weckstein JD, Takiya DM, Krejca JK, Murdoch JD, Veni G, Johnson KP, Reddell JR (2007). Phylogeography of cave crickets (*Ceuthophilus spp.*) in central Texas: A keystone taxon for the conservation and management of federally listed endangered cave arthropods.. *Illinois Natural History Survey Technical Report*.

[bibr12] U.S. Fish and Wildlife Service, Department of the Interior (1988). Endangered and Threatened Wildlife and Plants; Final rule to determine five Texas cave invertebrates to be endangered species.. *Federal Register*.

[bibr13] U.S. Fish and Wildlife Service, Department of the Interior (1993). Endangered and Threatened Wildlife and Plants; Coffin Cave Mold Beetle (*Batrisodes texanus*) and the Bone Cave Harvestman (*Texella reyesi*) determined to be endangered.. *Federal Register*.

[bibr14] U.S. Fish and Wildlife Service, Department of the Interior (2000). Endangered and Threatened Wildlife and Plants; Final rule to list nine Bexar County, Texas invertebrate species as endangered.. *Federal Register 65*.

[bibr15] Van Oosterhout C, Hutchinson WF, Wills DP, Shipley P (2004). Micro-checker: software for identifying and correcting genotyping errors in microsatellite data.. *Molecular Ecology Notes*.

[bibr16] Veni G, Elliott WR, Veni G (1994). Hydrogeology and evolution of caves and karst in the southwestern Edwards Plateau, Texas.. *The Caves and Karst of Texas.*.

[bibr17] Zane L, Bargelloni L, Patarnello T (2002). Strategies for microsatellite isolation: a review.. *Molecular Ecology*.

